# Efficacy of palliative stenting in patients with esophageal obstruction attributable to malignancy

**DOI:** 10.1002/deo2.70120

**Published:** 2025-04-22

**Authors:** Yasuki Hatayama, Hideaki Ishigami, Hidehiro Kamezaki, Daisuke Murakami, Yukiko Shima, Kentaro Ishikawa, Harutoshi Sugiyama, Takayoshi Nishino, Makoto Arai

**Affiliations:** ^1^ Department of Gastroenterology Tokyo Women's Medical University Yachiyo Medical Center Chiba Japan; ^2^ Department of Gastroenterology Chiba Rosai Hospital Chiba Japan; ^3^ Department of Gastroenterology Eastern Chiba Medical Center Chiba Japan

**Keywords:** cancer, esophageal stent, palliative care, prognosis, survival

## Abstract

**Background:**

Self‐expandable metallic stent (SEMS) placement is useful for patients with poor oral intake caused by esophageal stricture attributable to malignancy. In this study, we examined the usefulness of esophageal SEMS placement as a palliative treatment and evaluated the prognostic factors.

**Materials and methods:**

Patients who underwent esophageal SEMS placement at three regional base hospitals from December 2007 to June 2023 were included in the study.

**Results:**

Of 73 patients, 57 patients who underwent palliative SEMS placement were evaluated after excluding 16 patients in whom postoperative treatment was possible after SEMS placement. Median survival after SEMS placement was 67 days (mean, 96 ± 16 days). Univariate analysis identified age (≤78 years vs. >78 years), performance status (3 or 4 vs. 1 or 2), the cancer location (other sites vs. gastrointestinal cancer), the resumption of oral intake (failure vs. success), and clinical stage (IVA/IVB vs. III) as prognostic factors after SEMS placement. On multivariate analysis, performance status 3 or 4 (odds ratio [OR] = 2.87, 95% confidence interval [CI] = 1.28–6.45), cancers other than gastrointestinal cancer (OR = 3.75, 95% CI = 1.14–12.3), and failure to resume oral intake (OR = 21.3, 95% CI = 3.40–133.0) were significantly associated with poor prognosis.

**Conclusions:**

Palliative treatment with SEMS placement was safe, and a high percentage of patients resumed food intake. An inability to resume food intake, poor performance status, and cancer outside the gastrointestinal tract were poor prognostic factors.

## INTRODUCTION

Self‐expandable metallic stent (SEMS) placement is widely used in patients with esophageal stricture and poor oral intake.^1^ Endoscopic SEMS placement is less invasive and safe, and it is considered useful for both esophageal cancer and esophageal stricture caused by other types of cancer. Esophageal stenting can improve the quality of life in patients with advanced cancer by enabling oral intake in those in whom oral intake is prevented by gastrointestinal stenosis.^2^ Esophageal stent placement as described in the “Guidelines for the Diagnosis and Treatment of Esophageal Cancer” aims to improve symptoms caused by esophageal stricture and fistula as palliative care for patients in the terminal phase of their disease.^3^, ^4^ The indication for stenting after chemoradiotherapy is controversial because of the risk of perforation.^5^, ^6^, ^7^, ^8^, ^9^ There is also debate concerning whether chemoradiotherapy and other treatments can be safely performed after stenting.^10^, ^11^

In this study, we examined the usefulness of esophageal SEMS placement as a palliative treatment at three hospitals and evaluated the factors influencing prognosis.

## MATERIALS AND METHODS

### Patients and study design

A retrospective review of the medical records of all patients who underwent esophageal stent placement at three regional base hospitals in Japan between December 2007 and June 2023 was conducted. The present study was reviewed and approved by the institutional review board of Tokyo Women's Medical University (approval number 2022‐0005, 2023‐0070).

### SEMS procedures

Before stent placement, all patients or their relatives provided written informed consent. The patients were placed in a left lateral decubitus position on the fluoroscopy table. Patients were consciously sedated with midazolam and/or pentazocine during the procedure. Endoscopy was performed using an ultra‐thin endoscope (GIF‐XP290N; Olympus Medical Systems). The oral end of the stenosis was identified, and passage through the stenosis was first attempted using an endoscope. After the passage of the endoscope, the distance between the top and bottom of the stenosis was measured. If the passage of the endoscope was unsuccessful, then the length of the stenosis was measured under fluoroscopic guidance by injecting a contrast medium through the endoscopic channel using a catheter for endoscopic retrograde cholangiopancreatography. In this process, a guidewire was inserted through the endoscopic channel and passed through the stricture, and its tip was placed in the stomach or duodenum. The upper and lower margins of the tumor were identified fluoroscopically and marked on the fluoroscopy screen or body surface. The SEMS delivery device was inserted through the endoscopic channel along the guidewire, and SEMS placement was performed at the appropriate location. After stent deployment, the correct positioning of the stent was assessed endoscopically and radiographically.

### Outcome measurement

The clinical background characteristics of the patients (age, sex, primary site, clinical stage, and performance status [PS]) were analyzed. We assessed whether any complications related to SEMS placement had occurred, and evaluated the resumption of oral intake and the life expectancy after SEMS placement. The factors influencing prognosis were analyzed.

### Statistical analysis

The age and duration of observation for patients were presented as the mean ± SD. All analyses were performed using SPSS 26.0 (IBM Corp., Armonk, NY, USA). Univariate and multivariate analyses were performed to identify factors associated with prognosis. Factors identified as being significant by univariate analysis (*p* < 0.05) were entered into the model for multivariate logistic regression analysis. *p* < 0.05 denoted a statistically significant difference.

## RESULTS

### Patients’ clinical characteristics

In total 73 patients who underwent esophageal SEMS placement (83 procedures) at Tokyo Women's Medical University Yachiyo Medical Center, Chiba Rosai Hospital, and Eastern Chiba Medical Center in Chiba prefecture during the study period were identified. Indications for stent placement were judged comprehensively considering clinical stage, age, and comorbidities. The mean patient age was 74.8 ± 11.3 years, and 61 patients were male. The cancer types were esophageal cancer (*n* = 40), esophagogastric junction cancer (*n* = 24), lung cancer (*n* = 5), pancreatic cancer (*n* = 2, gastrointestinal stromal tumor (*n* = 1), and breast cancer (*n* = 1). Radiotherapy, chemotherapy, and surgery were performed prior to SEMS placement in two, 22, and nine patients, respectively. Because this study investigated SEMS placement as a palliative treatment, patients in whom active treatment (e.g., radiotherapy and chemotherapy) was provided after SEMS placement were excluded from further analysis. After excluding 16 patients in whom postoperative treatment was possible after SEMS placement, 57 patients who underwent SEMS placement as palliative treatment were selected for further analysis (Table 1). The numbers of patients at Tokyo Women's Medical University Yachiyo Medical Center, Chiba Rosai Hospital, and Eastern Chiba Medical Center were 30, 16, and 11, respectively. No cases of bleeding, perforation, fistula formation, or airway obstruction were observed in this study. In two patients, the stent was placed in a position that did not adequately resolve the stenosis caused by the tumor, requiring additional stent placement as a stent‐in‐stent. No instance of serious chest pain was recorded in the medical record. Complications observed during long‐term follow‐up included three cases of tumor invasion into the stent lumen or mouth or the anorectal side of the stent, which necessitated stent replacement, and two cases of restenosis attributable to intragastric fallout of the stent.

**TABLE 1:** Clinical characteristics of the patients.

	*n* = 57
Age (years) (average ± SD, range)	77.5 ± 9.2 (57–96)
Gender (male/female)	47/10
Performance status (1/2/3/4/undetermined)	9/12/19/1/16
Origin of cancer (esophagus/stomach/lung/mammary gland)	36/14/4/1/2
Brinkman Index (>400/<400/unknown)	30/9/8
Habitual alcoholic drinker (yes/no)	13/34
Observation period (days) (average ± SD, range)	90.7 ± 82.4(10–364)
Clinical stage(III/IVA/IVB)	14/13/30
Prior treatment (none/surgery/chemotherapy/radiation)	37/6/16/1
Details of the stent	
Length (15 cm/12 cm/10 cm/9 cm/8 cm/unknown)	9/19/12/1/13/3
Diameter (23 mm/22 mm/18 mm/16 mm/unknown)	1/3/49/1/3
Type (covered/uncovered/unknown)	46/5/6
Model (manufacturer)	
Niti‐S (Taewoong Medical Co., Ltd., Gyeonggi‐do, South Korea)/Ultraflex (Boston Scientific Co., Marlborough, MA, USA)/unknown	37/8/12

As reported previously,^12^ the dysphagia score was defined as follows: 0, “no dysphagia, able to eat a normal diet;” 1, “moderate passage, able to eat some solid foods;” 2, “poor passage, able to eat semi‐solid foods;” and 4, “very poor passage. able to swallow liquids only.” Food intake (dysphagia score ≤2) was possible in 54 patients (94.7%), and 43 patients (75.4%) could consume normal food (dysphagia score ≤1).

### Prognosis of patients after SEMS placement for palliative care

**FIGURE 1:**
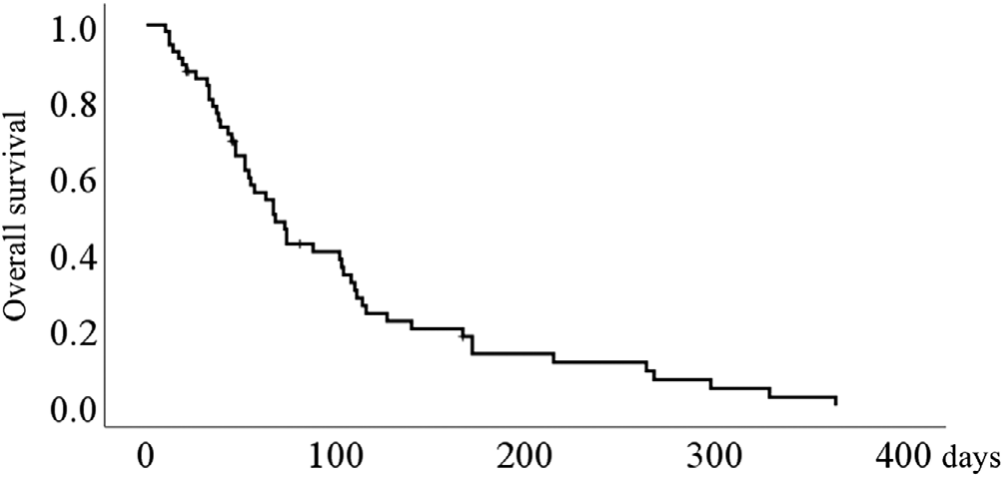
Survival curve for patients with esophageal obstruction attributable to malignancy after the palliative use of self‐expanding metallic stents. Mean and median survival were 100.3 ± 12.4 and 68 days, respectively.

The date of death was known in 51 of 57 patients. The mean duration of observation of patients whose date of death was known was 96.8 ± 81.9 days (range, 10–329), compared with 63.7 ± 55.1 days (range, 21–167) for patients whose date of death was unknown. The survival curve after SEMS placement is presented in Figure 1. The mean duration of survival was 100.3 ± 12.4 days (median, 68 days). Oral intake became possible in 27 patients (90%). Eighteen patients (60%) were able to consume an almost normal diet. There were no cases of serious intraoperative or postoperative complications.

### Factors affected prognosis after SEMS placement

**FIGURE 2:**
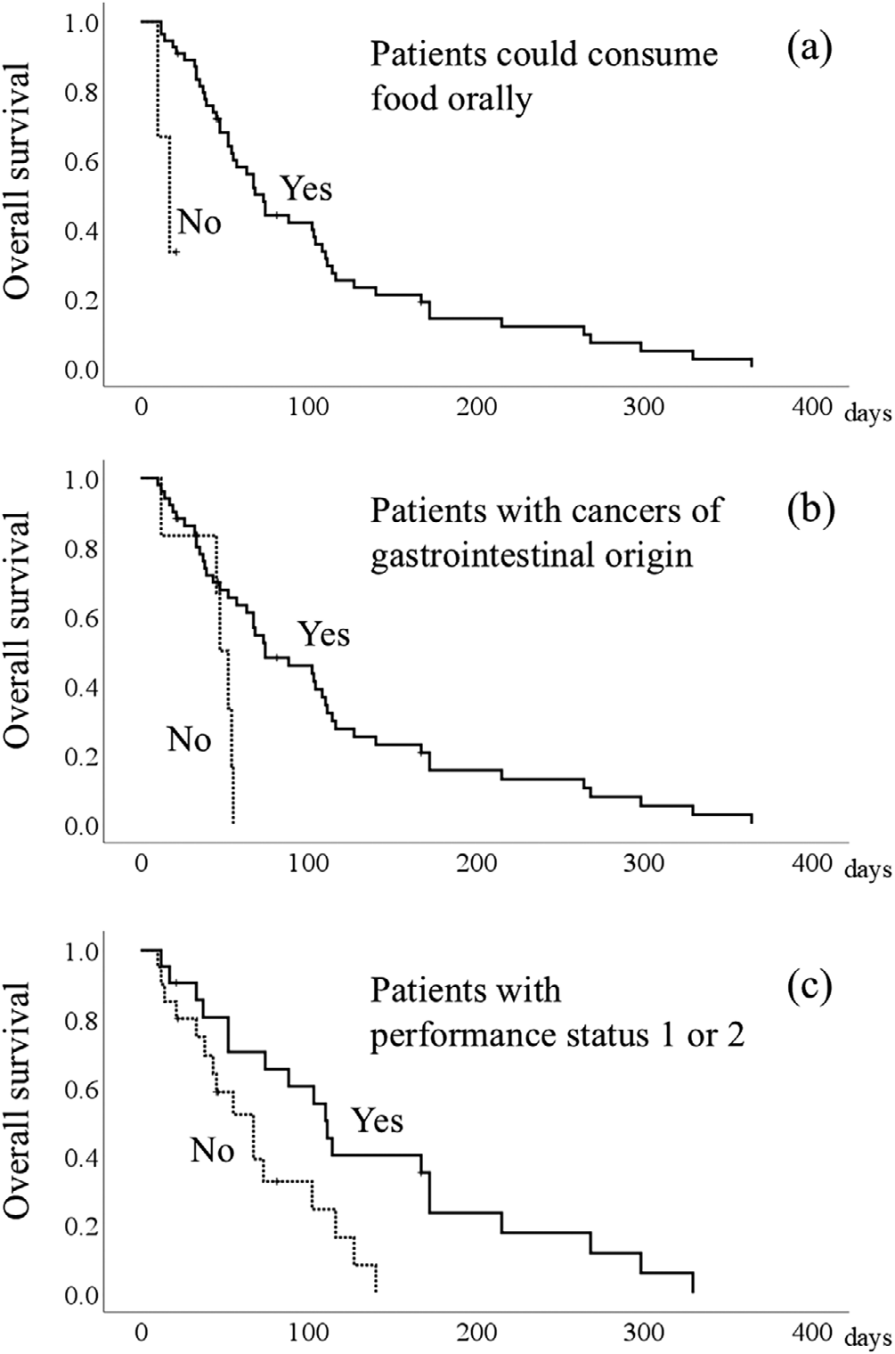
Survival curves after self‐expanding metallic stent placement according to different factors. (a) The mean survival time of patients who could consume food orally was 101.9 ± 16.4 days (solid line), versus 16.0 ± 2.6 days for those who could not resume oral intake (dotted line, log‐rank test *p* = 0.004). (b) The mean survival time of patients with cancers of gastrointestinal origin was 107.6 ± 13.6 days (solid line), compared with 44.2 ± 6.6 days for those with cancers of non‐gastrointestinal origin (dotted line, log‐rank test *p* = 0.011). (c) The mean survival time of patients with performance status 1–2 was 133.9 ± 21.9 days (solid line), versus 67.9 ± 10.3 days for those with performance status 2–4 (dotted line, log‐rank test *p* = 0.013.

In Table 2, the univariate analysis identified age (≤78 years vs. >78 years), PS (3 or 4 vs. 1 or 2), the cancer location (other sites vs. gastrointestinal cancer), the resumption of oral intake (failure vs. success), and clinical stage (IVA/IVB vs. III) as prognostic factors after SEMS placement. On multivariate analysis, PS 3 or 4, cancers other than gastrointestinal cancer, and failure to resume oral intake were significantly associated with poor prognosis. Although esophageal stents are intended to permit oral intake, oral intake was not achieved despite successful stent placement in a few patients. The mean survival of patients who could consume food orally was 101.9 ± 16.4 days (median, 88 days), versus 16.0 ± 2.6 days (median, 17 days) for those who did not achieve oral intake (log‐rank test *p* = 0.004, Figure 2a). Esophageal stents were used for cancers other than those of gastrointestinal origin in some cases. The prognosis of esophageal stenting was poorer in patients with cancers of non‐gastrointestinal origin than in those with cancers of gastrointestinal origin. Mean survival in patients with cancers of gastrointestinal origin was 107.6 ± 13.6 days (median, 68 days), compared with 44.2 ± 6.6 days (median, 49.5 days) for those with tumors that originated in other organs (log‐rank test *p* = 0.011, Figure 2b). Mean survival in patients with PS 0–1 was 133.9 ± 21.9 days (median, 110 days), versus 67.9 ± 10.3 days (median, 45.5 days) for those with PS 2–4 (log‐rank test *p* = 0.013, Figure 2c).

**TABLE 2:** Prognostic factors in patients with esophageal obstruction attributable to malignancy after self‐expanding metallic stent placement.

		Univariate analysis		Multivariate analysis	
Risk factor	Comparator	OR (95%CI)	*p*	OR (95%CI)	*p*
Age (≤78 years)	>78 years	2.19 (1.21–3.94)	0.009	1.82 (0.96–3.35)	n.s.
Gender (male)	Female	1.00 (0.50–2.01)	n.s.		
Performance status 3/4	Performance status 1/2	2.58 (1.19–5.59)	0.017	2.87 (1.28–6.45)	0.011
Organ (other than gastrointestinal tract)	Gastrointestinal tract	3.13 (1.23–7.69)	0.017	3.75 (1.14–12.3)	0.029
Brinkman index		1.00 (1.00–1.00)	n.s.		
Habitual alcoholic drinker		1.15 (0.57–2.32)	n.s.		
Resumption of oral intake (no)	Resumption of oral intake (yes)	11.7 (2.23–61.5)	0.004	21.3 (3.40–133.0)	<0.01
Esophagogastric junction location (yes)	Esophagogastric junction location (no)	0.68 (0.36–1.31)	n.s.		
Prior treatment (yes)	Prior treatment (no)	1.44 (0.80–2.61)	n.s.		
Clinical stage (IVB/IVA)	Clinical stage (III)	1.87 (1.06–3.31)	0.032	1.41 (0.76–2.63)	n.s.

Abbreviations: n.s., not significant; OR, odds ratio.

## DISCUSSION

Stent placement was performed safely in this study. Esophageal stents were implanted in 73 patients, but only 16 patients subsequently received aggressive treatment. In many cases, no aggressive treatment was given either before or after stent placement. Only a few patients required stent replacement because of restenosis or dropout after stent placement, and stent replacement was safely performed in these patients. Because this was a retrospective study, it was difficult to accurately assess minor mild symptoms such as chest pain. Severe symptoms were not noted in the medical record. To assess a patient's symptoms, it is necessary to conduct certain forms of questionnaire surveys. However, this is an issue for future study. A previous report indicated that patient survival was improved by repeat stenting in cases of tissue overgrowth.^13^ It was an excellent outcome that oral intake became possible in many cases without any safety issues. There have been many reports on the safety and usefulness of SEMS placement. Previous studies focused on factors such as the success rate of SEMS placement and demonstrated the benefit of SEMS placement for esophageal cancer or cancer of the esophagogastric junction, especially when used as palliative treatment. However, the relationship of SEMS placement with life expectancy has not been well‐studied. In addition, many reports about esophageal stent placement included patients who received aggressive treatment such as chemoradiotherapy after SEMS placement, and there have been no analyses of life expectancy in patients who underwent SEMS placement as palliative treatment. In Japan, a hyper‐aged society, patients are often diagnosed but not aggressively treated because of their advanced age and various complications.^14^ Many reports from Japan, where our study was conducted, described the usefulness of esophageal stents.^15^, ^16^, ^17^, ^18^ However, the long‐term prognosis of esophageal stent placement as a palliative treatment has not been fully evaluated, making our study extremely valuable. Therefore, it will become increasingly important to predict life expectancy in cases in which SEMS placement is used purely as a palliative treatment. Median survival after SEMS placement at a hospital in Kenya where chemoradiotherapy was unavailable was 250 days.^19^ The effectiveness of SEMS placement as a palliative treatment in areas where both radiotherapy and chemotherapy are available has been insufficiently studied. In the UK, the average life expectancy after SEMS placement was approximately 3 months,^20^ in line with our results (median, 100.3 ± 12.4 days; median, 68 days). The shorter survival compared with the results in Kenya is likely attributable to the various treatments used prior to SEMS placement. Information on how long oral intake can be maintained is of great importance to clinicians. Many patients are treated at the end of their lives in other hospitals or through home visits. If they are suspected of having difficulty eating because of restenosis, they are referred back to the specialized hospital. Therefore, for patients who were not referred back to the hospital, it was assumed that oral intake was maintained until death, but detailed information on the duration of oral intake was not available.

In the present study, we also included cancers other than those of digestive origin, whereas most previous reports focused only on cancers of gastrointestinal origin. Interestingly, the life expectancy after SEMS placement was significantly poorer for cancers of non‐digestive origin. For example, esophageal stenosis in patients with lung or breast cancer means that the tumor has invaded other organs, indicating more advanced disease. Elphick et al. reported that prognosis was poor for patients with respiratory malignancies, although the histological type and the exact esophageal location of the stricture did not influence prognosis.^21^ In general, tumor invasion from outside the gastrointestinal tract causes gastrointestinal fistula, making stents less effective.^22^ However, advances in chemotherapy have made it possible to control disease progression to some extent in cancers other than those of gastrointestinal origin. Consequently, we sometimes encounter cases in which general condition is maintained but they experience symptoms caused by narrowing of the gastrointestinal tract. The number of cases of gastrointestinal obstruction caused by metastasis or invasion into the gastrointestinal tract or by compression of the gastrointestinal tract attributable to perigastrointestinal lymph node metastasis is expected to increase in the future. Fortunately, even in cases of gastrointestinal stenosis attributable to cancers arising outside the gastrointestinal tract, SEMS placement was effective in our study.

A small number of patients were unable to ingest orally despite successful stenting. The prognosis of these patients was significantly worse, suggesting that although esophageal stenting physically allowed food to pass through the esophagus, the patients’ poor general condition was not associated with the inability to consume food orally. Thus, it is important to recognize that SEMS placement might not enable food intake. Mezes et al. reported that a large number of patients could not resume eating a regular diet but found no effect on life expectancy.^13^ In our study, patients who were unable to resume oral intake had a worse prognosis. The reasons for the different findings are unclear. Detailed evaluation of nutritional management after SEMS placement in patients who could not resume food intake has not been conducted, and further case accumulation is needed.

Stent placement can be safely performed even in older patients, and life expectancy was not related to age in this study. Comparisons were also made between different clinical stages, and although differences in the mean life expectancy were identified, the findings were not significant in multivariate analysis. In other words, esophageal stents enable oral intake and improve patients’ quality of life regardless of age or clinical stage. We did not directly assess patients’ quality of life in this study. Therefore, the effect of SEMS placement on patients’ quality of life cannot be described. However, the ability of SEMS placement to enable oral intake undoubtedly provided a benefit regarding quality of life. Thus, it can be presumed that most patients in this study experienced an improvement in quality of life. In addition, if oral intake is not possible, then inpatient care is generally required. However, if oral intake becomes possible, patients can be treated on an outpatient basis, which helps maintain social activities and improves their quality of life. Gastrostomy aims to improve the nutritional status of patients who cannot consume food orally because of esophageal obstruction. However, gastrostomy is difficult to perform in patients with large amounts of ascites or bleeding tendencies. The ability to consume food orally is extremely important for patients’ quality of life. Therefore, SEMS placement should be the first option in the palliative treatment of esophageal obstruction.

The prognosis of patients unable to resume oral intake after SEMS placement was extremely poor, suggesting that SEMS placement can have a negative impact on prognosis in certain cases. Although all patients who were unable to ingest orally after stenting had clinical stage IV cancer of gastrointestinal origin, the clinical characteristics examined in this study (e.g. age, PS, clinical stage, cancer of gastrointestinal origin) did not significantly differ between patients who could and could not resume oral intake after stenting (data not shown). In other words, it is extremely difficult to predict which patients will be unable to resume oral intake prior to SEMS placement. However, because SEMS placement carries a low physical burden and complications during SEMS placement are extremely rare, we believe that SEMS placement should be considered the first‐line treatment for esophageal obstruction.

## CONCLUSIONS

Regardless of age or clinical stage, SEMS placement is an effective treatment option or palliative therapy that should be aggressively considered in cases of esophageal stricture.

## CONFLICT OF INTEREST STATEMENT

We hereby state unequivocally that all fees for speaking at workshops co‐sponsored by pharmaceutical companies and grants from pharmaceutical companies are not directly related to this study, and that all companies were not involved in the planning, execution, or publication of this study.

Makoto Arai received lecture fees from Takeda Pharmaceutical Co., Otsuka Pharmaceutical Co., Ltd., AstraZeneca, Daiichi‐Sankyo Co., Bristol Myers Squib, MSD, Mitsubishi Tanabe Pharma Co., Taiho Pharmaceutical Co., ZERIA Pharmaceutical Co., Ltd., Chugai Pharmaceutical Co., ASKA Pharmaceutical Co., Ltd., Ono Pharmaceutical Co., Mochida Pharmaceutical Co. Ltd., Kowa Co., Ltd., Merck Biopharma Co., Ltd., AbbVie GK, Eli Lilly, Gilead Sciences, Inc.: contracted research grants from Chugai Pharmaceutical Co., AbbVie GK, Mochida Pharmaceutical Co. Ltd. The authors declare that they have no conflict of interest.

## ETHICS STATEMENT

Approval of the research protocol by an Institutional Reviewer Board: The present study was reviewed and approved by the institutional review board of Tokyo Women's Medical University (approval numbers 2022‐0005 and 2023‐0070).

## PATIENT CONSENT STATEMENT

The present study did not involve any invasion or intervention on the patient and used only information such as medical information. A number of the patients had either succumbed or had completed their hospital visits. The authors publicly announced the research plan and guaranteed the opportunity to refuse as much as possible. The ethical committee of Tokyo Women's Medical University permitted this opt‐out method. Therefore, informed consent was not achieved individually.

## CLINICAL TRIAL REGISTRATION

N/A.

## Data Availability

The data used and analyzed in this study are available from the corresponding author upon reasonable request.
